# Malaria infection confounds inflammation-adjusted micronutrient biomarker concentrations in children and women in Malawi: a secondary analysis of the 2015/2016 Malawi micronutrient survey

**DOI:** 10.1017/S0007114525000820

**Published:** 2025-05-14

**Authors:** Fanny Sandalinas, Edward J. M. Joy, Heidi Hopkins, Blessings H. Likoswe, Tineka Blake, Hanqi Luo, Melissa F. Young, Christian Bottomley, Parminder S. Suchdev, Suzanne Filteau

**Affiliations:** 1 Faculty of Epidemiology and Population Health, London School of Hygiene & Tropical Medicine, London, UK; 2 Faculty of Infectious and Tropical Diseases, London School of Hygiene & Tropical Medicine, London, UK; 3 Department of Public Health, School of Public Health and Family Medicine, College of Medicine, University of Malawi, Blantyre, Malawi; 4 School of Biosciences, University of Nottingham, Nottingham, UK; 5 Hubert Department of Global Health, Emory University, Atlanta, GA, USA; 6 Centers for Disease Control and Prevention, Atlanta, GA, USA

**Keywords:** Malaria, Micronutrient biomarkers, Ferritin, Folate, Inflammation

## Abstract

Inflammation and infections such as malaria affect concentrations of many micronutrient biomarkers and hence estimates of nutritional status. We aimed to assess the relationship between malaria infection and micronutrient biomarker concentrations in pre-school children (PSC), school-age children (SAC) and women of reproductive age (WRA) in Malawi and examine the potential role of malarial immunity on the relationship between malaria and micronutrient biomarkers. Data from the 2015/2016 Malawi micronutrient survey were used. The associations between current or recent malaria infection, detected by rapid diagnostic test and concentration of serum ferritin, soluble transferrin receptor (sTfR), zinc, serum folate, red blood cell folate and vitamin B_12_ were estimated using multivariable linear regression. Factors related to malarial immunity including age, altitude and presence of hemoglobinopathies were examined as effect modifiers. Serum ferritin, sTfR and zinc were adjusted for inflammation using the BRINDA method. Malaria infection was associated with 68 % (95 % CI 51, 86), 28 % (18, 40) and 34 % (13, 45) greater inflammation-adjusted ferritin in PSC, SAC and WRA, respectively (*P* < 0·001 for each). In PSC, the positive association was stronger in younger children, high altitude and children who were not carriers of the sickle cell trait. In PSC and SAC, sTfR was elevated (+ 25 % (16, 29) and + 15 % (9, 22) respectively, *P* < 0·001). Serum folate and erythrocyte folate were elevated in WRA with malaria (+ 18 % (3, 35) and + 11 % (1, 23), *P* = 0·01 and *P* = 0·003 respectively). Malaria affects the interpretation of micronutrient biomarker concentrations, and examining factors related to malarial immunity may be informative.

## Introduction

Micronutrient deficiencies underlie a large human disease burden, especially in low-income countries^(1)^, where infections are also common. The biochemical changes in a person’s body that are initiated in response to an infection, tissue injury or physiologic stressor are termed the inflammatory response^(2)^. A common feature is a rapid fall in the blood concentration of several micronutrients, including iron, zinc and retinol^(3)^. A reduction in micronutrient blood concentration, particularly iron, is believed to be beneficial to the host by depriving invading microorganisms of elements for growth and reproduction^(4)^. Although these depressions are transient and reversible, they have the potential to affect the accurate estimation of micronutrient status if the level of inflammation is high during the sample collection period, or if a large proportion of individuals with inflammation are sampled in a population survey.

### Changes in micronutrient biomarker concentrations associated with malaria

Methods exist to correct biomarker concentration for inflammation^(5)^. However, recent reports indicate that micronutrient biomarkers, especially ferritin, can be affected by malaria independently of inflammation^(6–9)^. This is of particular concern in sub-Saharan Africa, where iron deficiency and malaria tend to co-exist. Some research indicates that malaria might also affect the concentration of serum soluble transferrin receptors (sTfR)^(6,10)^, serum retinol^(7,11–13)^ and serum zinc^(14)^. The mechanisms are largely unknown but could involve the incomplete capture of the acute phase response by C-reactive protein (CRP) and *α*1-acid glycoprotein (AGP)^(12)^, increased erythropoiesis for sTfR^(10)^ and increased vitamin A requirements during malaria infection^(13)^ or micronutrient redistribution^(6)^. Other micronutrient biomarkers such as serum folate, erythrocyte folate and serum vitamin B_12_ seem not to be affected by inflammation^(15)^; however, there have been reports of elevated folate status in children during malaria infection, possibly due to the de novo synthesis of folate by the malaria parasite^(16)^. It is unknown if malaria infection results in elevated or modified folate and vitamin B_12_ status in women of reproductive age (WRA) and if these could affect estimates of folate and B_12_ deficiency prevalence to a degree that would impact decisions on implementation of programmes aimed at controlling these deficiencies.

## Naturally acquired malarial immunity

In previous studies analysing malaria and micronutrient biomarkers, it has been hypothesised that the relationship between malaria and micronutrient biomarkers could be modified by malarial immunity^(12,17,18)^. Naturally acquired immunity affects the likelihood of severe malaria in a given individual^(19)^. Naturally acquired antibody responses against *P. falciparum* require repeated parasite exposure to attain protection, and therefore, host immunity is determined by the total number of infections experienced by an individual, which is affected mainly by age and exposure^(20)^. Malaria transmission intensity (number of infectious bites per person per year) varies with a number of factors including altitude and environmental temperatures, which affect the development of *P. falciparum*^(21)^. A lower malaria transmission intensity in high-altitude regions results in lower malarial immunity among people residing in those regions. Similarly, urbanisation causes marked entomological, parasitological and behavioural changes that tend to result in reduced risks of malaria^(22)^. Additionally, the reduction in exposure associated with the use of mosquito nets could be associated with lower immunity to malaria^(23)^.

Hemoglobinopathies and enzymopathies such as sickle cell disease, *α*-thalassemia and glucose-6-phosphate dehydrogenase (G6PD) deficiency are common in sub-Saharan Africa^(24–26)^. While the genetic mutation that causes sickle cell disease can lead to early death in individuals who are homozygous for the mutation, in its heterozygous form (sickle cell carrier), it partially protects against severe malaria caused by *P. falciparum* infection^(27)^. Compared with persons with normal haemoglobin, individuals with sickle cell trait have a 50–90 % reduction in parasite density^(27)^. A number of mechanisms have been proposed, including reduced parasite growth and enhanced removal of parasitised cells through innate or acquired immune processes^(28)^. Alpha thalassemia is considered to be protective in cases of severe malaria but has no effect on asymptomatic parasitaemia^(29)^. G6PD-deficient alleles appear to confer a protective effect against malaria, although this protection seems to be limited to severe malaria^(30)^.

Identifying whether malarial immunity can modify the relationship between malaria and micronutrient biomarker concentrations should improve our understanding of the impact of malaria on micronutrient biomarkers interpretation and result in more accurate estimates of micronutrient status in individuals and populations.

## Natural malarial immunity and micronutrient biomarker concentrations in Malawi national micronutrient surveys

Malaria and iron deficiency have historically coexisted in Malawi^(31,32)^, as reported in previous national micronutrient surveys conducted in 2001 and 2009^(33,34)^. A national micronutrient survey was conducted in 2015 in order to report on the prevalence of micronutrient deficiencies in different population groups^(35)^. Although several analyses have focused on different factors impacting the level of micronutrient deficiencies in this survey^(36–38)^, the potential impact of malaria on the interpretation of micronutrient biomarker concentrations has not been analysed, even though the prevalence of malaria was found to be 28 % in pre-school children (PSC), 38 % in school-age children (SAC) and 17 % in WRA. In this analysis, we aimed to assess the relationship between malaria and micronutrient biomarker concentrations in these three population groups. We also aimed to examine the potential role of factors related to malarial immunity, such as age, altitude, rurality and presence of hemoglobinopathies, on the relationship between malaria and micronutrient biomarkers.

## Methods

### Data source

This analysis used data from the Malawi Micronutrient Survey, which was conducted in 2015–16^(35)^. Data were accessed from the Biomarkers Reflecting Inflammation and Nutritional Determinants of Anemia (BRINDA) group (https://www.brinda-nutrition.org/). The study design was reported in the Malawi Micronutrient Survey report^(35)^. Briefly, the Malawi Micronutrient Survey represented a subsample of the wider Demographic and Health Survey, which was designed as a cross-sectional study, with a two-stage cluster sampling design in order to obtain nationally representative indicators.

### Definition of variables

Deficiency in a specific micronutrient was defined as follows: Iron deficiency by inflammation-adjusted serum ferritin^(39)^ values below a cutoff of 12 μg/l in PSC and 15 µg/l in SAC and WRA^(9)^, zinc deficiency by inflammation-adjusted serum zinc^(39)^ values below a specific cutoff dependant on age, sex, fasting status and time of blood draw^(40)^, folate deficiency by values below a cutoff of 14 nmol/l for serum folate to define risk of elevated homocysteine and 748 nmol/l for erythrocyte folate to define risk of neural tube defects^(41)^ and vitamin B_12_ deficiency by values below 150 pmol/l for serum vitamin B_12_^(42)^. Serum folate, erythrocyte folate and vitamin B_12_ were available only for WRA.

High altitude was defined as an altitude above 1000 m based on literature^(43)^ and the fact that this was close to the median value of altitude in this survey. Principal component analysis was used in the Demographic and Health Survey survey to generate a wealth index in quintiles, based on the number and kinds of consumer goods they own. In the BRINDA dataset, the first and second quintiles were combined as ‘low socio-economic status (SES),’ the third and fourth quintiles were combined as ‘medium SES’ and the fifth quintile was considered as ‘high SES.’ Maternal education was categorised into four categories (some level of schooling, high school, at least 14 years of education, superior education). Whether the household owns a mosquito net for sleeping was asked in the Demographic and Health Survey questionnaire. The question did not provide details on whether it was used, in good condition, or if the children were sleeping under it.

The relationship between serum retinol and retinol binding protein had been tested in a subsample of participants, and the results showed a poor linear relationship between these two biomarkers, questioning the quality of measurement of either or both retinol binding protein and serum retinol^(36)^. We therefore decided not to include retinol binding protein in our analysis.

### Inflammation adjustment

For each dataset, ferritin, sTfR and zinc values were adjusted for inflammation using the regression approach with the BRINDA package^(39)^. A recent review showed weak and inconsistent correlations between CRP or AGP and vitamin B12 or folate biomarkers^(15)^, and therefore, we did not a priori adjust these biomarkers for inflammation. The regression approach has been described in detail elsewhere^(44)^ and uses linear regression to adjust biomarker concentrations by the CRP and AGP concentrations on a continuous scale. All the biomarker observations that had a corresponding CRP value and/or AGP value above the highest decile of the considered biomarker were adjusted with the linear regression. We used survey-specific internal deciles for this analysis to account for the context-specific pattern of inflammation. Although this is not yet recommended, we adjusted the biomarker values of ferritin, sTfR and zinc in SAC as well, as the correlations between biomarkers and CRP/AGP values were strong.

### Sample collection and laboratory analysis

Blood samples were collected in temporary central sites (field laboratories) through venipuncture. About 5 ml of whole blood was collected into a trace element-free vacutainer, from which serum was aliquoted for the analyses of ferritin, sTfR, CRP, AGP, retinol binding protein, zinc, folate and vitamin B_12_. Two millilitres of whole blood was also collected into ethylenediaminetetraacetic acid tubes for malaria detection and erythrocyte folate analysis, and 100 μl was stored as a dried blood spot for analysis of all hemoglobinopathies. All samples were kept in portable freezers in the field and transported to the nearest district laboratory for temporary storage at −20°C, before being transferred to the Community Health Sciences Unit (CHSU) where they were kept at −70°C as they awaited shipment.

Serum ferritin, sTfR, CRP and AGP were measured by sandwich ELISA at the VitMin lab (Willstaett, Germany)^(45)^. Zinc concentrations were analysed using Atomic emission spectrometry at the Children’s Hospital Oakland Research Institute (CHORI) in Oakland, USA. Serum folate and erythrocyte folate were measured with a microbiological assay (using *L. rhamnosus*) and vitamin B_12_ with an immunoassay in the CDC laboratory (Atlanta, USA). Malarial testing was done with an antigen-detecting rapid diagnostic test, the BIOLINE Malaria Ag *P.f*/Pan. Sickle cell, *α*-thalassemia and G6PD were diagnosed with PCR in the Cincinnati Children’s Hospital Medical Center, USA. Details of the PCR methods used have been described elsewhere^(46)^. These blood disorders were measured in PSC only to derive a ore accurate estimate of disease prevalence in Malawi, and as part of a government initiative to prepare a universal newborn screening program.

Quality control data were available for the analyses of ferritin, sTfR, zinc, CRP and AGP. The low, medium and high inter-assay CV for serum ferritin were 3·87 %, 3·39 % and 3·17 %, and for sTfR, it was 7·65 %, 5·45 % and 10·92 %, respectively. For CRP, the inter-assay CV was 7·36 %, 6·04 % and 4·21 % for low, medium and high reference material, and for AGP, it was 13·67 %, 11·76 % and 12·83 % for the same concentrations, respectively. Serum quality controls were from Bio-Rad laboratories. Certified reference serum seronorm level 1 and level 2 materials were used for the zinc analysis, and the CV for inter-assay error was 3·6 % for level 1 and 4·2 % for level 2.

Data on parasitaemia levels were not available. The presence of fever in the last 24 h was reported by the caregiver for PSC and SAC but not for WRA. Children were considered to be malaria symptomatic if they had a positive malaria test and a reported fever in the last 24 h. They were considered malaria asymptomatic if they had a malaria-positive test with no fever reported in the last 24 h.

### Statistical analysis

The outcome variables (ferritin, sTfR, zinc, folate, erythrocyte folate and vitamin B_12_) were continuous. The exposure variable, ‘malaria infection’, was binary (infected, uninfected). We examined the data to check for missing data, errors and inconsistencies and to gain an understanding of the distributions and patterns among the variables. Original sampling weights were used to describe the dataset and give nationally representative estimates of iron deficiency and malaria prevalence. Weights were not applied for the linear models or the measure of micronutrient status per infectious group, as the analyses were done to assess a biological association and were not supposed to be representative at any level. However, acknowledging that participants from the same cluster might have more similarities between them than participants from the entire sample, we conducted a sensitivity analysis using the clustering as a random effect in all linear models.

### Linear model

**Figure 1.**
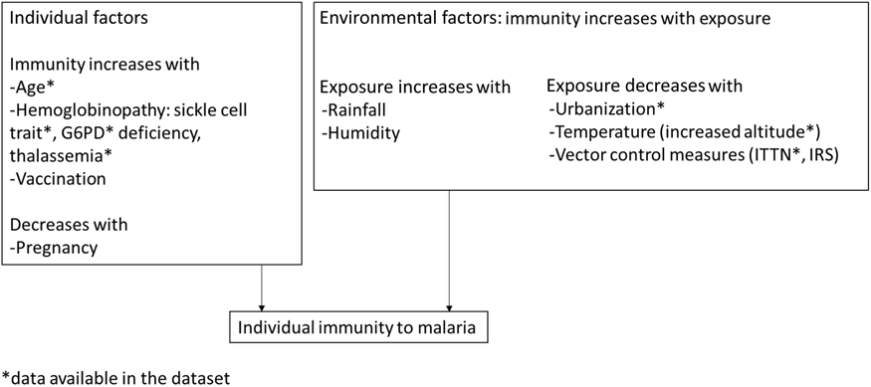
Identification of variables used to define malarial immunity in children and adults in the 2015 Malawi micronutrient survey. G6PD, glucose 6 phosphate dehydrogenase; ITTN, insecticide-treated nets; IRS, indoor residual spraying.

Multivariable linear regression analyses were conducted to estimate the association between malaria and micronutrient biomarker concentrations. The distribution of all micronutrient biomarker variables was skewed, and the log transformation (natural log) improved the original distributions. Consequently, the regression models were built on the logarithmic scale. If the estimated coefficient for malaria was *βˆ*, then a malaria infection was associated with a 100 × (*e ^β^*ˆ − 1) = per cent change in micronutrient biomarkers. The crude association between malaria and micronutrient biomarkers was assessed. The model was then adjusted for potential confounders: age (categorical, age group: < 2 years and ≥ 2 years for PSC, < 10 years and ≥ 10 years for SAC) or age in years for WRA, sex, rurality, socio-economic status, maternal or women’s education, deworming in the last 6 months, altitude and presence of sickle cell trait and *α*-thalassemia. The list of confounders was determined based on anticipated biological associations, particularly with regard to immunity to malaria (Figure 1). In WRA, information was available on the consumption of iron and folic acid supplements, and we included this variable in the model when analysing iron, serum and erythrocyte folate concentration, as the consumption of these supplements might have impacted iron and folate status. When analysing serum and erythrocyte folate concentrations, CRP and AGP were added to the model as correlations with these inflammatory markers were noticed. Two-factor interaction of each predictor variable with malaria infection was tested. The interactions with *P* > 0·1 were removed from the model. Interactions between malaria and rurality were not tested because of the very low number of cases of malaria in urban areas in all age groups. The coefficients from the linear model were used to calculate the malaria-adjusted biomarker concentrations and to estimate the prevalence of malaria-adjusted deficiency with the equation:

log(malaria-adjusted biomarker) = inflammation-adjusted biomarker + *βˆ*(malaria) + *βˆ_i_*(interactions).

When adding an interaction in the model, the main effect of each variable included in the interaction is also included in the model.

Model checking was based on residual and normal probability plots. All analyses were performed using R Statistical Software (2022.7.1.554; R Core Team 2022)^(47)^. Survey analyses account for the complex survey design, with the use of the ‘survey’ package^(48)^.

## Results

Micronutrient deficiencies affected all age groups, and zinc deficiency was the most common deficiency. SAC had a higher malaria prevalence than PSC or WRA (Table 1). 5·8 % of the PSC and 6·8% of the SAC were symptomatic.

**Table 1.** Characteristics from the 2015/2016 Malawi micronutrient survey participants (Mean values (or percentages) and 95 % CI)

	PSC (*n* 1233)			SAC (*n* 758)			WRA (*n* 776)		
	*n*	Mean (or %)	95 % CI	*n*	Mean (or %)	95 % CI	*n*	Mean (or %)	95 % CI
Age (mean)	1233	31·9	30·3, 33·4 months	758	9·6	9·4, 9·7 years	776	28·1	27·4, 28·9 years
Sex (% male)	1233	50·0	47·4, 53·0	758	50·9	47·3, 54·0	776	–	
Rurality (% rural)	1233	90·2	75·3, 97·0	758	87·7	80·1, 93·0	776	90·9	79·6, 96·0
Socio-economic status	1231			758			776		
Low (%)		50·2	44·2, 56·0		37·5	30·9, 45·0		42·7	35·5, 50·0
Medium (%)		40·5	36·3, 45·0		45·4	39·0, 52·0		44·5	38·2, 51·0
High (%)		9·3	5·7, 15·0		17·2	12·2, 24·0		12·8	7·9, 20·0
Positive malaria test (%)	1163	27·9	21·0, 36·0	750	37·6	31·9, 44	776	16·7	12·7, 22·0
Positive malaria test and fever in the last 24 h (%)	1156	5·8	3·8, 9·0	745	6·8	4·2, 11·0	Fever was not measured in WRA		
Iron deficiency (%)	1102	20·5	15·5, 26·7	758	4·5	3·3, 6·0	753	17·1	13·6, 21·0
Zinc deficiency (%)	1069	52·6	46·4, 59·0	746	39·4	33·8, 45·0	751	63·6	56·5, 70·0
Serum folate deficiency (%)	Not measured in PSC and SAC						748	34·2	28·3, 41·0
Erythrocyte folate insufficiency (%)							749	81·0	74·2, 86·0
Vitamin B_12_ deficiency (%)							757	13·0	9·3, 18·0
Carriers of sickle cell trait (%)	1074	9·1	6·5, 12·0		Not measured in SAC and WRA				
Carriers of alpha-thalassemia trait (%)	1070	43·0	37·6, 48·0						

PSC, pre-school children; SAC, school-age children; WRA, women of reproductive age. Indicators of micronutrient deficiencies were adjusted for inflammation with the BRINDA method^(5)^ and the following cutoffs were used: 12 µg/l for ferritin in PSC and 15 µg/l in SAC and WRA to define iron deficiency; specific cutoff for zinc depending on age, sex, fasting status and time of blood draw to define zinc deficiency; 14 nmol/l to define folate deficiency; 748 nmol/l to define red blood cell folate insufficiency; 150 pmol/l to define vitamin B_12_ deficiency. Data were weighted to account for survey design.

Inflammation-adjusted ferritin was significantly greater in individuals infected with malaria in all age groups (Table 2). In linear models, malaria was associated with 68 % relative higher inflammation-adjusted ferritin concentration in PSC, 28 % in SAC and 34 % in WRA (Table 2). In PSC and SAC, but not in WRA, sTfR was higher and zinc was slightly lower during malaria infection. In WRA, serum folate and erythrocyte folate were higher in people with a positive malaria rapid diagnostic test. The concentration of serum folate was negatively correlated with AGP, whereas erythrocyte folate concentration was not correlated with CRP nor AGP (online Supplementary Table 1). No difference in vitamin B_12_ concentrations was noted.

**Table 2.** Micronutrient biomarker concentrations by malaria infection in PSC (*n* 1163), SAC (*n* 749) and WRA (*n* 757) (geometric mean) from the 2015/2016 Malawi micronutrient survey (Percentages and 95 % CI)

	PSC					SAC					WRA				
	Uninfected (*n* 853)	Malaria-infected (*n* 310)	*P*^†^	Relative change		Uninfected (*n* 468)	Malaria-infected (*n* 281)	*P*^†^	Relative change		Uninfected (*n* 644)	Malaria-infected (*n* 113)	*P*^†^	Relative change	
				%	95 % CI				%	95 % CI				%	95 % CI
Serum ferritin (μg/l)*	20·9	37·3	<0·001	+ 67·6	50·7, 85·9	36·3	44·1	<0·001	+ 28·5	17·9, 40·3	28·1	36·0	<0·001	+ 34·0	14·4, 57·3
Serum sTfR (mg/l)*	7·7	9·5	<0·001	+ 25·2	17·3, 33·1	6·6	7·7	<0·001	+ 15·3	8·6, 22·4	6·6	6·9	0·3	No changes	
Serum zinc (μg/l)*	625	580	<0·001	–6·0	–9·6, −2·5	654	622	0·007	–5·0	–9·4, −0·4	591	577	0·1	No changes	
Serum folate (nmol/l)	Not measured in PSC and SAC										16·5	19·6	0·01	+ 17·6	2·6, 34·8
Erythrocyte folate (nmol/l)											498	580	0·003	+ 11·3	0·7, 22·9
Serum vitamin B_12_ (pmol/l)											289	301	0·5	No changes	

PSC, pre-school children; SAC, school-age children; WRA, women of reproductive age; sTfR, soluble transferrin receptors. *The concentrations of micronutrient biomarkers were adjusted for inflammation using the BRINDA method^(5)^. Serum zinc was not adjusted in WRA because of the weak correlation between zinc and CRP/AGP. ^†^Tests for the difference between groups were done on the log scale for all biomarkers, after adjustment for potential confounders (age, sex, rurality, socio-economic status, maternal or women’s education, deworming in the last 6 months, altitude and presence of sickle cell trait and alpha-thalassemia). In WRA, further adjustments were made for the consumption of iron and folic acid supplements for iron biomarkers, serum and erythrocyte folate and CRP and AGP for serum and erythrocyte folate concentrations). Adding the clustering as a random effect did not change the significance of the results.

Inflammation-adjusted ferritin was greater in PSC with symptomatic malaria compared with children with asymptomatic malaria. This was not the case in SAC (Table 3).

**Table 3.** Biomarker concentrations according to the stage of infection in PSC (*n* 1163) and SAC (*n* 745) (geometric mean) from the 2015/2016 Malawi micronutrient survey

	PSC				SAC			
	Uninfected (*n* 853)	Asymptomatic malaria (*n* 253)	Symptomatic malaria (*n* 57)	*P*^†^	Uninfected (*n* 467)	Asymptomatic malaria (*n* 244)	Symptomatic malaria (*n* 34)	*P*^†^
Serum ferritin (μg/l)*	20·9^a^	35·3^b^	48·0^c^	<0·001	36·3^a^	43·7^b^	44·6^b^	<0·001
Serum sTfR (mg/l)*	7·7^a^	9·8^b^	8·3^a^	<0·001	6·6^a^	7·7^b^	7·9^b^	<0·001
Serum zinc (μg/l)*	625^a^	597^b^	508^c^	<0·001	654	622	621	0·07

PSC, pre-school children; SAC, school-age children; WRA, women of reproductive age; sTfR, soluble transferrin receptors. *The concentrations of micronutrient biomarkers were adjusted for inflammation using the BRINDA method^(5)^. ^†^Tests for difference between groups were done on the log scale for all biomarkers, after adjustment for potential confounders (age, sex, rurality, socio-economic status, maternal education, deworming in the last 6 months, altitude and presence of sickle cell trait and *α*-thalassemia). Different letters indicate a statistical difference. Asymptomatic malaria was defined by a positive malaria test and the absence of fever in the last 24 h, as reported by the caregiver. Symptomatic malaria was defined by a positive malaria test and the presence of fever in the last 24 h, as reported by the caregiver.

### Impact of factors related to immunity

#### Ferritin

**Figure 2.**
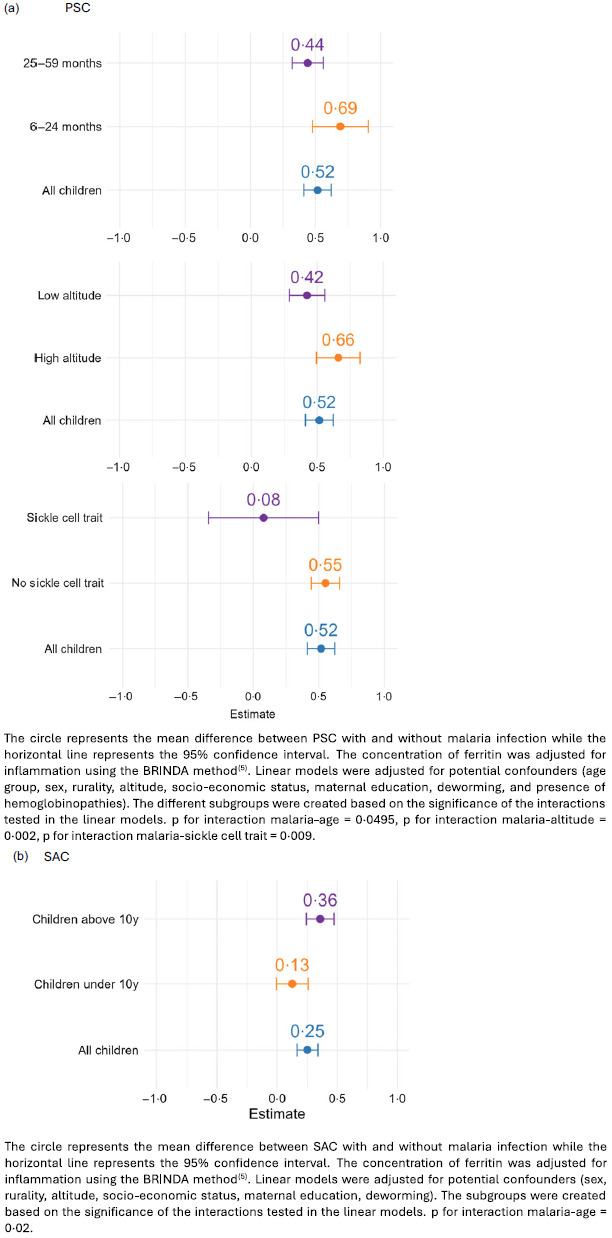
Difference in ferritin concentration (on the log scale) between pre-school (PSC) children with and without malaria infection ((a) *n* 1084) and school-age children (SAC) with and without malaria infection ((b) *n* 743) in different sub-groups of interest in the 2015/2016 Malawi micronutrient survey.

In PSC and SAC, but not in WRA, there was a significant interaction with age, as ferritin was less elevated during malaria infection in older children compared with young children (*P* for interaction = 0·0495 in PSC and 0·02 in SAC) (Figure 2(a) and (b)).

In PSC, there was also a significant interaction with altitude, as ferritin was greater at high altitude compared with low altitude in children with malaria (*P* for interaction = 0·002) (Figure 2). Being a carrier of sickle cell trait was also associated with a smaller difference in ferritin concentration during malaria in PSC (*P* for interaction = 0·009) (Figure 2). Being a carrier of the alpha-thalassemia trait was not associated with any differences in ferritin concentration in PSC infected with malaria.

#### Other micronutrient biomarkers

In PSC, sTfR were less elevated during malaria in high altitude compared with low altitude (*P* for interaction = 0·03) (online Supplementary Table 1). In PSC, being a carrier of the sickle cell trait was associated with a smaller reduction in serum zinc in children infected with malaria compared with non-carriers (*P* for interaction = 0·004) (online Supplementary Table 1). Factors related to immunity were not identified as effect modifiers in the relationship between malaria and zinc in SAC, nor between folate status and malaria in WRA.

Whether the household owned a mosquito net was not associated with any change in the relationship between malaria and inflammation-adjusted ferritin (data not shown).

Details of all linear models are presented in online Supplementary Table 1.

### Adjustment for malaria

No significant changes were noted in the prevalence of micronutrient deficiencies after adjusting for malaria (Table 4).

**Table 4.** Malaria adjustment impact on micronutrient deficiencies in three population groups (PSC, *n* 1084; SAC, *n* 743; WRA, *n* 753) from the 2015/2016 Malawi micronutrient survey (Percentages and 95 % CI)

	Inflammation-adjustment only		Inflammation and malaria adjustment	
	Prevalence in %	95 % CI	Prevalence in %	95 % CI
PSC				
Iron deficiency	20·5	15·5, 27·0	23·8	19·5, 29·0
Zinc deficiency	52·6	46·4, 59·0	49·4	43·5, 55·0
SAC				
Iron deficiency	4·5	3·3, 6·0	5·1	3·5, 7·0
Zinc deficiency	39·4	33·8, 45·0	35·9	30·7, 41·0
WRA				
Iron deficiency	17·1	13·6, 21·0	18·4	15·0, 22·0
Zinc deficiency	63·6	56·5, 70·0	63·6	56·5, 70·0
Folate deficiency (serum folate)	34·2	28·3, 41·0	36·5	30·3, 43·0
Erythrocyte folate insufficiency	81·0	74·2, 86·0	82·2	75·8, 87·0
Vitamin B_12_ deficiency	13·0	9·3, 18·0	13·0	9·3, 18·0

PSC, pre-school children; SAC, school-age children; WRA, women of reproductive age; Indicators of micronutrient deficiencies were adjusted for inflammation with the BRINDA method^(5)^ and the following cutoffs were used: 12 μg/l for ferritin to define iron deficiency in PSC and 15 μg/l in SAC and WRA, specific cutoff for zinc dependent on age, sex, fasting status and time of blood draw to define zinc deficiency, 14 nmol/l to define folate deficiency, 746 nmol/l to define red blood cell folate insufficiency and 150 pmol/l to define vitamin B_12_ deficiency. Data were weighted to account for survey design. The adjustment for malaria was made using coefficients from a linear regression that included the main effect of malaria and the interactions found significant for each population group and for each indicator (for PSC: model B for ferritin, model F for zinc; for SAC: model G for ferritin, model J for zinc; for WRA: model K for ferritin, model L for serum folate, model M for erythrocyte folate, online Supplementary Table 1).

### Relationship between indicators of iron status

Malaria can affect the two indicators of iron status, ferritin and sTfR, in different ways. By adjusting for malaria and therefore by at least partially removing the effect of malaria on these indicators, we expect a better relationship between these indicators. Adjusting for malaria resulted in a stronger correlation between sTfR and ferritin in PSC (Table 5). The malaria adjustment did not modify the relationship between ferritin and sTfR in WRA.

**Table 5.** Coefficient of correlation (Spearman test) between indicators of iron status in PSC (*n* 1084), SAC (*n* 743) and WRA (*n* 753) from the 2015/2016 Malawi micronutrient survey

	Inflammation-adjusted ferritin	Malaria and inflammation-adjusted ferritin
PSC		
Inflammation-adjusted sTfR	–0·27 (*P* < 0·001)	
Malaria and inflammation-adjusted sTfR		–0·37 (*P* < 0·001)
SAC		
Inflammation-adjusted sTfR	–0·08 (*P* = 0·02)	
Malaria and inflammation-adjusted sTfR		–0·13 (*P* < 0·001)
WRA		
Inflammation-adjusted sTfR	–0·45 (*P* < 0·001)	
Malaria and inflammation-adjusted sTfR		–0·46 (*P* < 0·001)

PSC, pre-school children; SAC, school-age children; WRA, women of reproductive age; sTfR, soluble transferrin receptors. Indicators of micronutrient deficiencies were adjusted for inflammation with the BRINDA method^(5)^. Correlations were only tested between ferritin and sTfR if both variables were either malaria-adjusted or non-malaria-adjusted. The adjustment for malaria was made using coefficients from a linear regression that included the main effect of malaria and the interactions found significant for each population group and each indicator (For PSC: model B for ferritin, model F for zinc; for SAC: model G for ferritin, model J for zinc; for WRA: model K for ferritin, model L for serum folate, model M for erythrocyte folate, online Supplementary Table 1).

## Discussion

In our analysis of the data from the 2015/16 micronutrient survey in Malawi, serum ferritin, sTfR, zinc, folate and erythrocyte folate were affected by current or recent malaria infection in the population groups studied and factors related to immunity modified the relationship between malaria and iron biomarkers in children.

The difference in ferritin between individuals with and without malaria infection was greater in PSC compared with SAC and WRA. It was also greater in PSC with symptomatic malaria compared with PSC with asymptomatic malaria. In PSC and SAC, a greater difference in ferritin was seen in younger children compared with older children. The apparent absence of the effect of factors related to immunity on ferritin concentration during malaria in WRA could be due to the fact that immunity is already acquired by adulthood, although this would only explain the lack of age effect. In PSC, altitude was also found to be an effect modifier: during malaria infection, ferritin elevations were greater at higher altitudes compared with lower altitudes. Altitude is known to be a limiting factor for the reproduction of *P. falciparum*,^(21,49)^ and this finding could result from lower immunity to malaria among children living in high altitudes.

In children who carry the sickle cell trait, malaria was associated with a lower concentration of ferritin compared with children infected with malaria who were not sickle cell trait carriers. It has been shown that the presence of the sickle cell trait is associated with a 50–90 % reduction of the parasite density^(26)^, which could explain why ferritin is lower during malaria in children with the sickle cell trait^(27)^. It has been reported previously that sickle cell trait distribution was not homogenous in Malawi, with a very low prevalence in the south of the country^(50)^. This geographical distribution could result in iron deficiency prevalence being particularly under-estimated in the south of the country, where ferritin is likely to be more elevated during malaria compared with areas with a higher prevalence of sickle cell disease. Neither being a carrier of the alpha thalassemia trait nor being deficient in G6PD was associated with a lower decrease in ferritin during malaria, which is consistent with earlier findings, as it has been shown that *α*-thalassemia and G6PD deficiency do not protect against asymptomatic malaria^(29)^.

sTfR concentrations were higher in children with malaria but to a lesser extent at high altitudes. This seems contradictory as there is an increase in erythropoiesis during malaria and, in high altitudes, we should expect a further increase in sTfR. Conflicting findings on sTfR in malaria have been reported in the past^(10,51,52)^. The haemolysis associated with malaria infection could increase sTfR concentrations by stimulating erythropoiesis, but there is also evidence of inhibition of erythropoiesis during acute malaria infection, which would be expected to decrease sTfR concentrations^(53)^. In Malawi, cases of malaria could be more acute at high altitudes because of lower immunity, which could explain the reduction in sTfR at high altitudes.

### Implications for the assessment of iron status

The results from this analysis indicate that particular attention should be given to children with characteristics hypothesised to be associated with a low immunity to malaria when assessing their iron status, whether this is at the individual level or when analysing population-level data. Adjusting for malaria resulted in small, non-significant differences in micronutrient deficiency prevalence. However, even small differences could be of public health importance, especially if policymakers are relying on thresholds to define the severity of micronutrient deficiencies or are using these data for cost-effectiveness analyses to decide on the allocation of spending. Moreover, identifying the factors that modify the relationship between serum ferritin and malaria can help to identify in which settings a malaria adjustment would be necessary, for example, in areas of high altitude and in young children. The fact that the iron indicators were more closely correlated after the malaria adjustment in PSC and SAC seems to indicate that this adjustment results in a more accurate estimation of iron status. The fact that malaria is associated with increased ferritin, even after adjusting for inflammation should be considered especially when ferritin is analysed as a continuous variable, for example, when assessing the efficacy of an intervention to improve iron status. Elevated ferritin concentrations in children with characteristics hypothesised to be associated with low immunity to malaria were probably associated with elevated hepcidin, resulting in a blockage of iron absorption, and could help to understand why micronutrient interventions are not always effective in improving population micronutrient status in apparently healthy individuals^(54,55)^. More attention could be given to other infections that could have an inflammation-independent effect on micronutrient status, such as norovirus^(56)^.

### Implications for other biomarkers

Malaria infection could be considered when reporting the folate status of the population. Even though the changes in biomarkers associated with malaria infection were modest in our analyses, the impact might be different in other contexts. Malaria was associated with higher serum and erythrocyte folate, while inflammation was associated with lower serum folate. This suggests an inflammation-independent effect of malaria on folate metabolism. Furthermore, both serum and erythrocyte folate were affected to the same extent, which could indicate both a short and long-term effect of malaria on folate status. This is, to our knowledge, the first time this has been reported in a national micronutrient survey. Population-level data on folate status are important to evaluate public health policies and programmes such as folic acid fortification. The elevation of folate during malaria has been previously observed in children and is believed to be due to either de novo folate synthesis by the pathogen, altered folate utilisation in infected erythrocytes or reticulocytosis^(16)^. In settings where malaria prevalence is higher than in this survey, the impact on folate status could be important.

Reductions in plasma zinc concentrations have been reported in uncomplicated acute malaria^(14)^ but not in asymptomatic malaria infections^(12)^. The small but similar reduction in both PSC and SAC serum zinc is consistent with earlier findings. Many factors are considered when interpreting indicators of zinc status, particularly the fact that serum zinc is not considered a very sensitive indicator of zinc status^(40)^, and the small reductions that we observed in our analysis do not seem to justify particular attention to malaria status when assessing zinc status with serum zinc. Further analysis in other contexts could help determine the impact of malaria on serum zinc.

### Strengths and limitations

One of the major strengths of the study is the use of data from a large nationally representative survey with individual data on multiple micronutrients. There is also a consistency of findings with regard to factors related to immunity. All of the factors included in this analysis that are considered to correlate with malarial immunity (age, altitude, sickle cell trait) modified the relationship between malaria and ferritin in the same direction. The results were consistent across age groups and biomarkers in children.

One of the limitations of the study is that there was no measure of malarial immunity. More attention may be given to the measurement of malaria antibodies and hepcidin in individuals to better understand how malarial immunity may modify iron metabolism during a malaria infection. Another limitation of this study is the impossibility of determining whether relationships were causal since the data were cross-sectional. However, prospective studies on malaria infection and indicators of iron status^(57–59)^ suggested a causal and positive effect of malaria on ferritin levels. Additionally, the difference in inflammation–ferritin concentration between asymptomatic and symptomatic malaria cases suggests that the changes in ferritin are dependent on the severity of malaria infection, which also suggests a causal mechanism.

Malaria infections were detected by rapid diagnostic test, and further analyses could be necessary to confirm that similar findings are observed in infections detected by other methods (e.g. microscopy or PCR, which may be used in other surveys). We also note that some of the effects were small, and the confidence limits were wide.

### Conclusion

Our analysis showed that malaria infection in PSC, SAC and WRA was associated with some changes in micronutrient biomarker concentrations, even after controlling for inflammation with CRP and AGP. The relationship between malaria and ferritin was the strongest and the most consistent across all age groups. Women with malaria infection had significantly higher serum folate and erythrocyte folate than those uninfected. Malarial immunity-related factors were identified as modifying the relationship between ferritin and malaria. More attention may be important to give to children with factors hypothesised to be associated with low immunity to malaria, such as high altitude, young age and absence of sickle cell trait, when assessing their iron status in malaria-endemic areas.

## Supporting information

Sandalinas et al. supplementary material Sandalinas et al. supplementary material

## Supplementary material

For supplementary material/s referred to in this article, please visit https://doi.org/10.1017/S0007114525000820
