# Supplementary material for: Malaria infection confounds inflammation-adjusted micronutrient biomarker concentrations in children and women in Malawi: a secondary analysis of the 2015/2016 Malawi micronutrient survey
Source: Br J Nutr. 2025 Apr 11;133(9):1276–86. doi: 10.1017/S0007114525000820 (PMC12229982; doi:10.1017/S0007114525000820)
Supplement: Sandalinas et al. supplementary material [file S0007114525000820sup001.docx]

**Supplementary Table 1**: Results of the final linear model for each biomarker for each population group (PSC, n=1,084; SAC, n=743; WRA, n=753) from the 2015/2016 Malawi micronutrient survey

Pre-school children, PSC (n=1,084)

|  | **Difference in log ferritin (95% IC)** | **p value** |
| --- | --- | --- |
| **Model A:** log ferritin in PSC (main effect) | | |
| Malaria (infected vs non-infected) | 0.52 (0.41, 0.62) | <0.001 |
| Age group  (< 2y vs > 2y) | -0.66 (-0.75, -0.56) | <0.001 |
| Sex (girls vs boys) | 0.06 | 0.2 |
| Altitude (high vs low) | -0.02 | 0.6 |
| Rurality (rural vs urban) | 0.14 | 0.09 |
| Socio-economic status (ascending socio-economic status) | -0.02 | 0.7 |
| Maternal education (ascending education) | 0.004 | 0.9 |
| Deworming in the last 6 months (yes vs no) | -0.03 | 0.6 |
| Sickle cell carrier (carrier vs non-carrier) | -0.005 | 0.9 |
| Alpha-thalassemia (carrier vs non carrier) | -0.06 | 0.2 |
| R^2^:0.26  P<0.001 |  | |
| **Model B:** log ferritin in PSC (including interactions) | | |
| Malaria (infected vs non-infected) | 0.36 (0.21, 0.51) | <0.001 |
| Age group  (< 2y vs >2y) | -0.71 (-0.82, -0.60) | <0.001 |
| Sex (girls vs boys) | 0.05 | 0.3 |
| High altitude (high altitude vs low altitude) | -0.1 | 0.06 |
| Rurality (rural vs urban) | 0.13 | 0.09 |
| Socio-economic status (ascending socio-economic status) | -0.02 | 0.6 |
| Maternal education (ascending education) | 0.006 | 0.9 |
| Deworming in the last 6 months (yes vs no) | -0.04 | 0.6 |
| Sickle cell trait (carrier vs non carrier) | 0.08 | 0.3 |
| Alpha-thalassemia (carrier vs non carrier) | -0.05 | 0.3 |
| Interaction  Malaria*age group (< 2y vs >2y) | 0.22 (0.001, 0.44) | p for the interaction  0.0495 |
| Interaction  Malaria*high altitude (high altitude versus low altitude) | 0.33 (0.12, 0.53) | p for the interaction  0.002 |
| Interaction  Malaria*sickle cell trait (carrier vs non carrier) | -0.51 (-0.89, -0.13) | p for the interaction  0.009 |
| R^2^:0.27  P<0.001 |  | |

|  | **Difference in log sTfR (95% IC)** | **p value** |
| --- | --- | --- |
| **Model C:** log sTfR in PSC (main effect) | |  |
| Malaria (infected vs non-infected) | 0.23 (0.16, 0.29) | <0.001 |
| Age group  (< 2y vs >2y) | 0.38 (0.32, 0.44) | <0.001 |
| Sex (girls vs boys) | -0.04 | 0.1 |
| Altitude (high vs low) | 0.0002 | 0.9 |
| Rurality (rural vs urban) | 0.02 | 0.7 |
| Socio-economic status (ascending socio-economic status) | 0.06 (0.02, 0.11) | 0.004 |
| Maternal education (ascending education) | 0.001 | 0.9 |
| Deworming in the last 6 months (yes vs no) | -0.03 | 0.4 |
| Sickle cell carrier (carrier vs non-carrier) | 0.06 | 0.1 |
| Alpha-thalassemia (carrier vs non carrier) | 0.05 | 0.08 |
| R^2^:0.20  P<0.001 |  |  |
| **Model D:** log sTfR in PSC (including interactions) | |  |
| Malaria (infected vs non-infected) | 0.28 (0.20, 0.35) | <0.001 |
| Age group  (< 2y vs >2y) | 0.38 (0.32, 0.43) | <0.001 |
| Sex (girls vs boys) | -0.04 | 0.1 |
| High altitude (high altitude vs low altitude) | 0.03 | 0.3 |
| Rurality (rural vs urban) | 0.02 | 0.7 |
| Socio-economic status (ascending socio-economic status) | 0.06 (0.02, 0.11) | 0.003 |
| Maternal education (ascending education) | -0.0007 | 0.9 |
| Deworming in the last 6 months (yes vs no) | -0.03 | 0.4 |
| Sickle cell trait (carrier vs non carrier) | 0.07 | 0.1 |
| Alpha-thalassemia (carrier vs non carrier) | 0.04 | 0.1 |
| Interaction  Malaria*high altitude (high altitude versus low altitude) | -0.13 (-0.25, -0.01) | p for the interaction  0.03 |

|  | **Difference in log zinc (95% IC)** | **p value** |
| --- | --- | --- |
| **Model E:** log zinc in PSC (main effect) | | |
| Malaria (infected vs non-infected) | -0.07 (-0.10, -0.03) | 0.001 |
| Age group  (< 2y vs >2y) | 0.02 | 0.4 |
| Sex (girls vs boys) | 0..02 | 0.3 |
| Altitude (high vs low) | 0.02 | 0.2 |
| Rurality (rural vs urban) | 0.06 | 0.06 |
| Socio-economic status (ascending socio-economic status) | 0.03 | 0.05 |
| Maternal education (ascending education) | 0.02 | 0.3 |
| Deworming in the last 6 months (yes vs no) | 0.007 | 0.7 |
| Sickle cell carrier (carrier vs non-carrier) | 0.02 | 0.4 |
| Alpha-thalassemia (carrier vs non carrier) | 0.02 | 0.2 |
| R^2^:0.02  P<0.001 |  | |
| **Model F:** log zinc in PSC (including interactions) | | |
| Malaria (infected vs non-infected) | -0.08 (-0.12, -0.04) | 0.0001 |
| Age group  (< 2y vs >2y) | 0.02 | 0.3 |
| Sex (girls vs boys) | 0.02 | 0.2 |
| High altitude (high altitude vs low altitude) | 0.02 | 0.3 |
| Rurality (rural vs urban) | 0.06 (0.0004, 0.12) | 0.049 |
| Socio-economic status (ascending socio-economic status) | 0.03 (0.001, 0.06) | 0.04 |
| Maternal education (ascending education) | 0.02 | 0.2 |
| Deworming in the last 6 months (yes vs no) | 0.008 | 0.7 |
| Sickle cell trait (carrier vs non carrier) | -0.01 | 0.7 |
| Alpha-thalassemia (carrier vs non carrier) | 0.02 | 0.2 |
| Interaction  Malaria*sickle cell (carrier vs non carrier) | 0.21 (0.07, 0.36) | p for the interaction  0.004 |
| R^2^:0.03  P<0.001 |  | |

School-age children, SAC (n=743)

|  | **Difference in log ferritin (95% IC)** | **p value** |
| --- | --- | --- |
| **Model F:** log ferritin in SAC (main effect) | | |
| Malaria (infected vs non-infected) | 0.25 (0.16, 0.34) | <0.001 |
| Age in years (over 10y vs under 10y) | -0.04 | 0.4 |
| Sex (girls vs boys) | 0.003 | 0.9 |
| Altitude (high vs low) | 0.1 (0.03, 0.19) | 0.008 |
| Rurality (rural vs urban) | -0.11 | 0.1 |
| Socio-economic status (ascending socio-economic status) | -0.05 | 0.2 |
| Maternal education (ascending education) | 0.09 (0.01, 0.16) | 0.02 |
| Deworming in the last 6 months (yes vs no) | 0.005 | 0.9 |
| R^2^:0.07  P<0.001 |  | |
| **Model G:** log ferritin in SAC (including interactions) | | |
| Malaria (infected vs non-infected) | 0.14 (0.01, 0.27) | 0.03 |
| Age in years (increasing age) | -0.11 (-0.22, -0.009) | 0.03 |
| Sex (girls vs boys) | 0.004 | 0.9 |
| High altitude (high altitude vs low altitude) | 0.10 (0.02, 0.19) | 0.01 |
| Rurality (rural vs urban) | -0.11 | 0.1 |
| Socio-economic status (ascending socio-economic status) | -0.04 | 0.2 |
| Maternal education (ascending education) | 0.08 (0.003, 0.15) | 0.04 |
| Deworming in the last 6 months (yes vs no) | 0.01 | 0.9 |
| Interaction  Malaria*age (above 10y vs below 10y) | 0.19 (0.03,0.36) | p for the interaction  0.02 |
| R^2^:0.08  P<0.001 |  | |

|  | **Difference in log sTfR (95% IC)** | **p value** |
| --- | --- | --- |
| **Model H:** log sTfR in SAC (main effect) | | |
| Malaria (infected vs non-infected) | 0.14 (0.08, 0.20) | <0.001 |
| Age (above 10 vs below 10) | -0.02 | 0.6 |
| Sex (girls vs boys) | 0.02 | 0.5 |
| Altitude (high vs low) | -0.04 | 0.2 |
| Rurality (rural vs urban) | 0.04 | 0.4 |
| Socio-economic status (ascending socio-economic status) | 0.003 | 0.9 |
| Maternal education (ascending education) | -0.01 | 0.6 |
| Deworming in the last 6 months (yes vs no) | 0.003 | 0.9 |
| R^2^:0.05  P<0.001 |  | |

|  | **Difference in log zinc (95% IC)** | **p value** |
| --- | --- | --- |
| **Model J:** log zinc in SAC (main effect) | | |
| Malaria (infected vs non-infected) | -0.05 (-0.1, -0.004) | 0.03 |
| Age in years (increasing age) | 0.002 | 0.7 |
| Sex (girls vs boys) | 0.03 | 0.3 |
| Altitude (high vs low) | 0.005 | 0.8 |
| Rurality (rural vs urban) | 0.1 (0.04, 0.2) | 0.004 |
| Socio-economic status (ascending socio-economic status) | 0.03 | 0.07 |
| Maternal education (ascending education) | -0.007 | 0.7 |
| Deworming in the last 6 months (yes vs no) | 0.01 | 0.8 |
| R^2^:0.01  P=0.06 |  | |

Women of reproductive age, WRA (n=753)

|  | **Difference in log ferritin (95% IC)** | **p value** |
| --- | --- | --- |
| **Model K:** log ferritin in WRA (main effect) | | |
| Malaria (infected vs non-infected) | 0.29 (0.13, 0.44) | <0.001 |
| Age in years (increasing age) | 0.004 | 0.2 |
| Socio economic status (increasing socio-economic status) | 0.001 | 0.9 |
| Maternal education (increasing education) | -0.008 | 0.9 |
| High altitude (high altitude versus low altitude) | 0.14 (0.03, 0.25) | 0.01 |
| Rurality (rural against urban) | 0.17 (-0.34, -0.01) | 0.04 |
| R^2^:0.03  P<0.001 |  | |

|  | **Difference in log serum folate (95% IC)** | **p value** |
| --- | --- | --- |
| **Model L:** log serum folate in WRA (main effect) | | |
| Malaria (infected vs non-infected) | 0.16 (0.03, 0.30) | 0.02 |
| Age in years (increasing age) | -0.003 | 0.3 |
| Socio economic status (increasing socio-economic status) | -0.11 (-0.19, -0.04) | 0.003 |
| Education (increasing education) | -0.10 (-0.19, -0.01) | 0.03 |
| Iron supplementation (took iron vs did not take iron) | -0.01 | 0.9 |
| Rurality (rural against urban) | 0.17 (0.02, 0.31) | 0.02 |
| High altitude (high altitude versus low altitude) | 0.24 (0.15,0.34) | <0.001 |
| CRP (increasing CRP) | -0.004 (-0.01, 0.003) | 0.3 |
| AGP (increasing AGP) | -0.2 (-0.3, -0.01) | 0.04 |
| R^2^:0.11  P<0.001 |  | |

CRP: C-reactive protein, AGP: α1-acid glycoprotein

|  | **Difference in log red blood cell folate**  **(95% IC)** | **p value** |
| --- | --- | --- |
| **Model M:** log red blood cell folate in WRA (main effect) | | |
| Malaria (infected vs non-infected) | 0.11 (0.07, 0.21) | 0.04 |
| Age in years (increasing age) | 0.0005 | 0.8 |
| Socio economic status (increasing socio-economic status) | -0.02 | 0.5 |
| Education (increasing education) | -0.07 (-0.13, -0.005) | 0.03 |
| Iron supplementation (took iron vs did not take iron) | 0.29 (0.03, 0.55) | 0.03 |
| Rurality (rural against urban) | 0.12 (0.01, 0.22) | 0.03 |
| High altitude (high altitude versus low altitude) | -0.09 (-0.16, -0.02) | 0.008 |
| CRP (increasing CRP) | -0.002 (-0.01, 0.003) | 0.4 |
| AGP (increasing AGP) | -0.02 (-0.3, -0.01) | 0.7 |
| R^2^:0.04  P<0.001 |  | |
